# Clinical Characteristics of *Corynebacterium* Bacteremia Caused by Different Species, Japan, 2014–2020

**DOI:** 10.3201/eid2712.210473

**Published:** 2021-12

**Authors:** Ryosuke Yamamuro, Naoto Hosokawa, Yoshihito Otsuka, Ryosuke Osawa

**Affiliations:** Kameda Medical Center, Chiba, Japan

**Keywords:** Corynebacterium striatum, Corynebacterium jeikeium, contamination rate, bacteremia, hematologic malignancy, bacteria, Japan

## Abstract

To determine differences in clinical characteristics of patients with bacteremia caused by *Corynebacterium striatum*, *C. jeikeium*, and other species of *Corynebacterium*, we retrospectively reviewed medical records of patients in Japan who had *Corynebacterium* bacteremia during January 2014–May 2020. Of the 115 records evaluated, 60 (52%) were cases of true bacteremia and 55 (48%) were cases of contamination. Proportions of true bacteremia cases caused by *C. striatum* (70%) and by *C. jeikeium* (71%) were significantly higher than those caused by other species of *Corynebacterium* (9%). These 2 organisms were commonly detected in blood cultures of patients with hematologic malignancies and neutropenia. The mortality rates at 90 days were 34% (*C. striatum*), 30% (*C. jeikeium*), and 0 (other species). Given the high mortality rates, assessing true bacteremia when *C. striatum* or *C. jeikeium* is detected in blood cultures, especially in patients with hematologic malignancy, is warranted.

## Abstract 2

*Corynebacterium* bacteremia is most commonly caused by *C. striatum* or *C. jeikeium*.

*Corynebacterium* bacteria are club-shaped gram-positive rods that are ubiquitous in the environment. Because *Corynebacterium* species other than *C. diphtheriae* colonize skin and mucous membranes in humans, *Corynebacterium* is typically considered a clinically nonsignificant contaminant in cultures (*1*). Recently, the frequency of detecting *C. striatum* and *C. jeikeium* as causative agents of severe bloodstream infections (*2*,*3*), infective endocarditis, pneumonia, meningitis, and skin and soft tissue infections (SSTIs) has increased (*4*). Furthermore, these 2 species have been identified most frequently in cultures of clinical specimens, mainly blood, pus, urine, and pleural effusion (*5*).

Studies that have identified *Corynebacterium* infections or bacteremia to the species level are limited, and most are case reports (*6*). The largest study to date of *Corynebacterium* bacteremia investigated 98 cases; however, the species were not identified (*7*). The largest study that identified *Corynebacterium* species included 30 cases of true bacteremia in 339 patients with positive blood cultures (*8*). In our study, we aimed to determine the differences in characteristics and clinical presentations for patients with bacteremia caused by *C. striatum*, *C. jeikeium*, or other species of *Corynebacterium*.

## Materials and Methods

### Study Design

We retrospectively reviewed electronic medical records and clinical microbiology records of patients with positive blood cultures for *Corynebacterium* spp. in Kameda Medical Center (Chiba, Japan) during January 2014–May 2020. This facility is an 865-bed, tertiary-care general medical center that provides a wide variety of services including general medicine, surgery, oncology, cardiothoracic surgery, hematopoietic stem cell transplantation, and renal transplantation to ≈310,000 persons each year. All patients with blood cultures positive for any organism are automatically referred to the infectious diseases department for consultation. Board-certified infectious disease physicians evaluate the patients and document the consultation report in medical records. The study protocol was reviewed and approved by the Kameda Medical Center Institutional Review Board (reference no. 20-046). The need for written informed consent was waived. The study complied with the principles of the Declaration of Helsinki.

### Study Population

We included all patients at the hospital who had blood cultures positive for *Corynebacterium* spp. during the study period. We collected data about age, sex, underlying conditions, clinical diagnosis, 90-day mortality rates, species of *Corynebacterium*, and antimicrobial susceptibility. If the same patient had multiple episodes of *Corynebacterium* bacteremia during the study period, we included only the first episode.

### Definitions

We defined a case as true bacteremia when 2 sets of blood cultures from a patient with signs of infection were positive for *Corynebacterium* spp. or when 1 set of blood cultures and a clinically relevant specimen from another site (e.g., urine or sputum) where the infection was thought to exist (on the basis of signs/symptoms and examination findings) were both positive for the same species of *Corynebacterium*. For patients with only 1 set of blood cultures in which *Corynebacterium* spp. were detected and for whom bacteremia was clinically suspected, new blood cultures were performed, and reevaluated as necessary, before antimicrobial agents were initiated. These patients were carefully followed by our infectious disease physicians to ensure the absence of infection. This definition was based on a previous study (*9*).

Catheter-related bloodstream infection (CRBSI) was considered definite for patients who met 1 of the following 3 criteria: 1) >1 set of blood cultures and semiquantitative cultures of a catheter segment (>15 CFUs/plate) were both positive for the same *Corynebacterium* species; 2) peripheral blood cultures and blood cultures from a catheter lumen were both positive for the same species of *Corynebacterium*, and its differential time to positivity was >2 hours (*10*); or 3) 2 sets of blood cultures were positive for *Corynebacterium* species, and signs of inflammation or purulence were present at the catheter insertion site (*11*). Diagnosis of other focal infections were based on the US Centers for Disease Control and Prevention National Healthcare Safety Network criteria (*12*).

We classified a case as no focus when physical examination by infectious disease physicians revealed no localized signs of infection, urinalysis was negative for pyuria or bacteriuria, chest images (radiographs or computed tomography scans) showed no infiltrates or masses, and the case still satisfied the criteria for true bacteremia. Chronic kidney disease was defined as being present when serum creatinine level was >2.0 mg/dL. Liver disease was defined as presence of liver cirrhosis or chronic hepatitis B or C.

### Laboratory Methods

We used RapID CB Plus (Kyokuto Pharmaceutical Industrial Co. Ltd., https://www.kyokutoseiyaku.co.jp) for bacterial identification during January 2014–May 2015. This kit correctly identifies 95% of *Corynebacterium* isolates to the species level (*13*). Starting in June 2015, we identified strains by using matrix-assisted laser desorption/ionization time-of-flight mass spectrometry and a Bruker MALDI Biotyper (Bruker Daltonics GmbH, https://www.bruker.com). We used score cutoff values according to recommendations proposed by the manufacturer (>2.0). For some cases in which no identification or ambiguous identification was achieved by these methods, we confirmed identification by using 16S rRNA gene sequence analysis. We performed antimicrobial susceptibility tests by broth microdilution, using Clinical and Laboratory Standards Institute (CLSI, https://clsi.org) M45 A2:2ED 2010 during January 2014–December 2016 and CLSI M45 3rd edition from January 2017 on.

### Statistical Analyses

We used Fisher exact or Pearson χ^2^ tests to compare categorical variables. For continuous variables, we used Mann–Whitney U or paired *t*-tests, and for estimating survival probabilities we used Kaplan-Meier curves. We estimated and compared the cumulative incidence of mortality by using the log-rank test and compared differences in antimicrobial susceptibility between *Corynebacteria* species by using Fisher exact or Pearson χ^2^ tests. We considered p<0.05 to indicate statistical significance. We performed all statistical analyses by using EZR (Saitama Medical Center, Jichi Medical University, Saitama, Japan), a graphical user interface for R (The R Foundation, https://www.r-project.org) (*14*).

## Results

### Proportion of True Bacteremia Cases

Of 115 patients in this study, *C. striatum* was detected in 67 (58%), *C. jeikeium* in 14 (12%), and other *Corynebacteria* species in 34 (30%) patients. The category of other consisted of 15 species (Table 1). In total, there were 60 cases of true bacteremia and 55 cases of contamination, resulting in 52% of patients having true bacteremia. Of the 60 patients with true bacteremia, 55 had >2 sets of positive blood cultures with *Corynebacterium* spp.; 5 had 1 set of positive blood cultures but met the definition of true bacteremia in our study. Of 115 patients, >2 genera of bacteria were detected in blood culture from only 1 patient; this patient had diverticulitis and bacteremia caused by *Corynebacterium* spp. and *Escherichia coli*. The patient recovered after receiving treatment for *E. coli* bacteremia alone; *Corynebacterium* spp. were considered to be contaminants. The percentages of true bacteremia cases caused by *C. striatum* (70%) and *C. jeikeium* (71%) were significantly higher than those for other species (9%; p<0.001 for each) (Table 2).

**Table 1:** Patients with *Corynebacterium* species detected in blood cultures, Japan, 2014–2020

Corynebacterium species	Total, n = 115	True bacteremia, n = 60	Contamination, n = 55
*C. striatum*	67	47	20
*C. jeikeium*	14	10	4
Other, total	34	3	31
* C. accolens*	1	0	1
* C. afermentans*	6	0	6
* C. amycolatum*	4	1	3
* C. aurimucosum*	4	0	4
* C. coyleae*	1	0	1
* C. glucuronolyticum*	1	0	1
* C. minutissimum*	4	0	4
* C. mucifaciens*	1	0	1
* C. pseudodiphtheriticum*	1	0	1
* C. resistens*	2	0	2
* C. riegelii*	1	1	0
* C. simulans*	3	0	3
* C. singulare*	2	0	2
* C. tuberculostearicum*	2	0	2
* C. urealyticum*	1	1	0

**Table 2:** Clinical diagnosis and characteristics of patients with *Corynebacterium* species detected in blood culture, Japan, 2014–2020*

Variable	All, n = 115	*C. striatum*, %, n = 67	*C. jeikeium*, %, n = 14	Other species, %, n = 34	p values	
					*C. striatum* vs. other species	*C. jeikeium* vs. other species
Age, y	71	71	66	77	0.055	<0.001
Sex						
** M**	80 (70)	51 (76)	13 (93)	16 (47)	<0.01	<0.001
F	35 (30)	16 (24)	1 (7)	18 (53)	<0.01	<0.001
Underlying disease, no. (%)						
** Diabetes mellitus**	27 (23)	14 (20)	2 (14)	11 (32)	0.309	0.292
** Chronic kidney disease**	16 (14)	9 (13)	2 (14)	5 (15)	1	1
** Liver disease**	4 (4)	4 (6)	0 (0)	0 (0)	0.299	NA
** Solid tumor**	27 (24)	13 (19)	4 (29)	10 (29)	0.378	1
** Leukemia**†	20 (17)	11 (16)	8 (57)	1 (3)	0.056	<0.001
** Malignant lymphoma**‡	14 (12)	8 (12)	3 (21)	3 (9)	0.537	0.171
** Hematologic malignancy**§	38 (33)	24 (36)	9 (64)	5 (15)	0.036	<0.01
Underlying condition, no. (%)						
Neutropenia, <500 cells/mm^3^	29 (25)	19 (28)	8 (57)	2 (6)	<0.01	<0.001
** Corticosteroid **	8 (7)	5 (8)	2 (14)	1 (3)	0.661	0.2
** Chemotherapy, within 3 mo**	41 (36)	23(34)	10 (71)	8 (24)	0.377	<0.01
Clinical diagnosis, no. (%)						
** True bacteremia**	60 (52)	47 (70)	10 (71)	3 (9)	<0.001	<0.001
** No focus**	25 (22)	19 (29)	6 (43)	0	ND	ND
** CRBSI**	17 (15)	13 (19)	3 (21)	1 (3)	ND	ND
** Other focus**¶	18 (16)	15 (22)	1 (7)	2 (6)	ND	ND
** Contamination**	55 (48)	20 (30)	4 (29)	31 (91)	<0.001	<0.001

*ALL, acute lymphoid leukemia; AML, acute myeloid leukemia; CRBSI: catheter-related blood stream infection; DLBCL, diffuse large B cell lymphoma; MDS, myelodysplastic syndrome; MM, multiple myeloma; NA, not applicable; ND, not done; IVLBCL, intravascular large B cell lymphoma.; PTCL, peripheral T-cell lymphoma.

†No. cases: AML, 17; ALL, 3,

‡No.cases: DLBCL, n = 8: PCTL, n =3; IVLBCL, n = 1; lymphoplasmacytic lymphoma, n= 1,: Burkitt lymphoma, n = 1.

§No. cases: leukemia, n = 20; lymphoma, n = 14; MM, n = 3; MDS, n = 1; myelofibrosis, n = 2.

¶No. cases: pyelonephritis, n = 5; skin and soft tissue infection, n = 4; empyema, n = 2; pneumonia, n = 1; prostatitis, n = 1; infective endocarditis, n = 1; osteomyelitis, n = 1; vascular graft infection, n = 1; central venous port infection, n = 1; spontaneous bacterial peritonitis, n = 1.

### Clinical Diagnosis and Underlying Diseases

Hematologic malignancy was the most common underlying disease (33%), especially in 64% of patients with *C. jeikeium* bacteremia, followed by solid tumors (24%) and diabetes mellitus (23%) (Table 2). *C. striatum* and *C. jeikeium* were more frequently detected than other species in patients with hematologic malignancy (p = 0.036 and p<0.001, respectively) and neutropenia (p<0.01 and p<0.001, respectively). Of the 60 patients with true bacteremia, 25 (42%) had infection at an unknown site; 17 (28%) had CRBSI; and 18 (30%) had infection at other foci, including SSTI, pyelonephritis, pneumonia, empyema, infective endocarditis, vertebral osteomyelitis, central venous port infection, and spontaneous bacterial peritonitis.

### Mortality Rates

Mortality rates among patients with true bacteremia were 34% among those with bacteremia caused by *C. striatum*, 30% by *C. jeikeium*, and 0 by other species of *Corynebacterium*. (Figure). We observed no significant differences in survival rates between these groups (*C. striatum* p = 0.25 and *C. jeikeium* p = 0.32). Six patients experienced a fulminant course of illness that resulted in death within 7 days; for all 6 patients, the causative organism was *C. striatum*.

**Figure:**
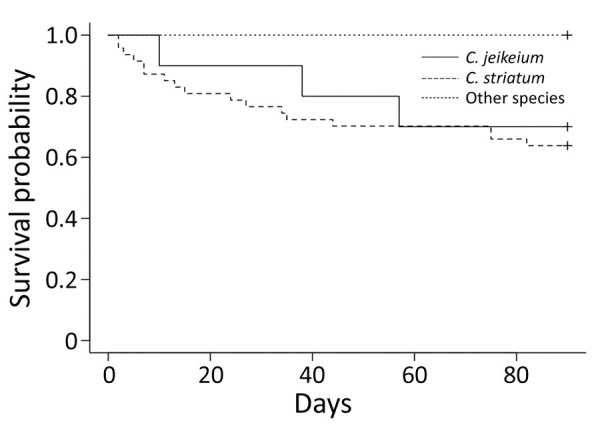
Kaplan-Meier curve showing survival probability after episodes of true bacteremia caused by *Corynebacterium* species, Japan, 2014–2020.

### Antimicrobial Susceptibility

All tested strains of *Corynebacterium*, regardless of species, were susceptible to vancomycin, linezolid, and minocycline (Table 3). *C. striatum* and *C. jeikeium* were less susceptible than other species to penicillin (p<0.001 for each), ceftriaxone (p<0.001 for each), meropenem (p<0.001 for each), erythromycin (p<0.01 for each), and ciprofloxacin (p<0.001 for *C. striatum* and p = 0.02 for *C. jeikeium*).

**Table 3:** Antimicrobial susceptibility testing of *Corynebacterium* species isolated from blood culture, Japan, 2014–2020*

Species	Susceptible/tested (%)									
	PEN	CRO	MEM	GEN	CIP	MIN	CLI	ERY	VAN	LZD†
*C. striatum*, n = 67	14/67 (21)	5/67 (7)	17/67 (25)	59/67 (88)	3/67 (4)	67/67 (100)	8/67 (12)	13/67 (19)	67/67 (100)	4/4 (100)
*C. jeikeium*, n = 14	0/14 (0)	0/14 (0)	5/14 (36)	5/14 (36)	0/14 (0)	14/14 (100)	0/14 (0)	0/14 (0)	14/14 (100)	3/3 (100)
Other species, n = 34	27/34 (79)	22/34 (65)	31/34 (91)	30/34 (88)	10/34 (29)	34/34 (100)	6/34 (18)	16/34 (47)	34/34 (100)	2/2 (100)
All, n = 115	41/115 (36)	27/115 (23)	53/115 (46)	94/115 (82)	13/115 (11)	115/115 (100)	14/115 (12)	29/115 (25)	115/115 (100)	9/9 (100)

*CIP, ciprofloxacin; CLI, clindamycin; CRO, ceftriaxone; ERY, erythromycin; GEN, gentamicin; LZD, linezolid; MEM, meropenem; MIN, minocycline; PEN, penicillin; VAN, vancomycin.

†Antimicrobial susceptibility testing for linezolid was only performed if requested by physicians.

## Discussion

With regard to the characteristics of *Corynebacterium* bacteremia at the species level, we report 3 major findings. First, *C. striatum* and *C. jeikeium* each caused true bacteremia more frequently than did other *Corynebacterium* species. Second, hematologic malignancies were the most common underlying disease in patients with *Corynebacterium* bacteremia (33%). Third, although the most common sources of infection were of unknown origin and CRBSI, other sources (e.g., pyelonephritis, SSTI, and empyema) accounted for 30% of true bacteremia cases.

The strengths of our study include having had infectious disease specialists assess infection sites and classify cases as true bacteremia to ensure study quality. Furthermore, detailed clinical and microbiological data were available because we included all cases of *Corynebacterium* bacteremia in our center over 6 years.

A previous study reported that contamination rates varied among species of *Corynebacterium* and that *C. jeikeium* caused true bacteremia more frequently than other species (*8*). The overall contamination rates of 48% in all patients treated in our study approximated those for 2 previous studies in Japan (46% and 42%) (*9*,*15*). However, higher contamination rates were reported in a study performed in Sweden (*8*), where 93% of these cases were considered to be contaminations and *C. afermentans* accounted for 14%, *C. aurimucosum* for 7%, and *C. amycolatum* for 6% of the total *Corynebacterium* species detected in blood culture. Our study also demonstrated a high contamination rate of 93% for those species, but our frequency of detection was less than that in the previous study; detection rates in our study were 5% for *C. afermentans*, 3% for *C. aurimucosum*, and 3% for *C. amycolatum*. The difference in contamination rates in both studies may be underpinned by regional differences in the epidemiology of *Corynebacterium* species. It is plausible that the study populations may differ because the study in Sweden was population based, whereas our study was performed in a tertiary-care hospital. Furthermore, indications for blood culture may differ between these studies

Bacteremia with *C. striatum* or *C. jeikeium*, the most frequently identified species in our study, seemed to be more associated with a higher 90-day mortality rate when compared with other species, although we observed no significant difference. Factors associated with a poor prognosis for *Corynebacterium* spp. bacteremia are mixed infection, chronic kidney disease, and lack of a central venous catheter (*16*). However, we are unaware of any study that has reported on differences in mortality rate among patients with infections by different species of *Corynebacterium*. One study reported that *C. striatum* formed biofilms on polyurethane catheters in vitro and hypothesized that biofilm may contribute to the establishment of hospital-acquired infections (*17*). Indeed, biofilm formation has been associated with true bacteremia in another study (*18*). *C. jeikeium* has also been reported to form biofilm, which can promote opportunistic infections (*19*). Although further study is needed, the tendency for *C. striatum* and *C. jeikeium* to form biofilm and their association with true bacteremia may be a reason for worse outcomes compared with outcomes for infection with other *Corynebacterium* species.

All strains of *Corynebacterium* spp. detected in this study were sensitive to vancomycin, minocycline, and linezolid. A previous study reported that most isolates were resistant to penicillin, ciprofloxacin, and tetracycline, and in contrast, all isolates were sensitive to vancomycin (*20*). In another study, only a few strains of *C. jeikeium* were resistant to doxycycline (*21*). The results of our study are consistent with those reports.

The most common underlying disease in patients with *Corynebacterium* bacteremia in our study was hematologic malignancy (33%). Among bacteremic patients with hematologic malignancies, the second most common gram-positive bacteria were *Corynebacterium* spp. (*22*). *C. striatum* was more likely to cause bacteremia in patients with malignancies or neutropenia (*15*), and *C. jeikeium* also caused bacteremia, frequently in patients with neutropenia or a history of previous antimicrobial treatment (*23*). The reason for the higher frequency of *Corynebacterium* bacteremia in patients with hematologic malignancies remains unknown. Among patients with hematologic malignancies, the reported rate of skin or rectal colonization with *Corynebacterium* spp. was 41% (*24*). We hypothesize that skin and mucosal barrier failures resulting from intense chemotherapy, chronic indwelling infusion catheters, and increased colonization may put patients with hematologic malignancies at a higher risk for *Corynebacterium* bacteremia.

Although the most common sources for *Corynebacterium* bacteremia were unknown or CRBSI, other sources accounted for 30% (18/60 cases), including lower respiratory tract infections, urinary tract infections, and SSTIs. Previous studies have reported that *C. striatum* can cause pneumonia (*25*), urinary tract infections, and intra-abdominal infections (*4*). Case studies have also reported *C. jeikeium* as being responsible for infective endocarditis (*6*), pacemaker infections (*26*), and prosthetic joint infections (*27*). *Corynebacterium* spp. are often reported as coryneform and not fully identified unless they are from sterile specimens because they colonize the skin and are ubiquitous in the environment. We emphasize the value of actively identifying coryneforms in specimens, even if they are not sterile (e.g., sputum or urine), especially in suspected cases of *Corynebacterium* bacteremia.

The first limitation of our study is that it was a retrospective single-center study. However, we believe that our results can be generalized to other tertiary institutions because the common species of *Corynebacterium* and susceptibility results obtained in our study do not differ considerably from others (*9*,*20*); moreover, our hospital is a referral center providing tertiary care in the region. Second, because of the difficulty of separating true bacteremia from contamination when *Corynebacterium* spp. are detected in blood culture, it is possible that we may have missed patients with true bacteremia. For example, we may have missed a patient with prosthetic valve endocarditis when *Corynebacterium* spp. were detected in only 1 set of blood cultures as a result of previous antimicrobial drug use because it did not meet the criteria for true bacteremia in our study. It is also possible that detection of *Corynebacterium* in 2 sets of blood cultures may actually represent contamination. To minimize the risk of incorrectly categorizing *Corynebacterium* bacteremia into true bacteremia or contamination, each case was carefully discussed during daily rounds and cases were routinely closely followed up with repeated blood culture if deemed necessary. Third, the number of cases of infection with the other 15 species of *Corynebacterium* was small, and the sample size was insufficient to describe the clinical characteristics of bacteremia caused by each of these species. Last, we used RapID CB Plus and matrix-assisted laser desorption/ionization time-of-flight mass spectrometry mainly for identification and performed 16s rRNA sequencing analysis for only a subset of cases.

In conclusion, the proportion of cases of true bacteremia caused by *C. striatum* or *C. jeikeium* was higher than that caused by other *Corynebacterium* species, and the mortality rate for true bacteremia was ≈30%. *C. striatum* and *C. jeikeium* were frequently detected in patients with hematologic malignancies and neutropenia. Healthcare providers should give special consideration to these 2 species of *Corynebacterium* and consider the possibility of true bacteremia rather than contamination when they are detected in blood cultures, especially in patients with hematologic malignancies.

## References

[1] von Graevenitz A, Pünter-Streit V, Riegel P, Funke G. Coryneform bacteria in throat cultures of healthy individuals. J Clin Microbiol. 1998;36:2087–8. 10.1128/JCM.36.7.2087-2088.1998 9650969 PMC104985

[2] Elkayam N, Urazov A, Tuneev K, Chapnick E. *Corynebacterium* striatum bacteremia associated with cellulitis in a patient with cirrhosis. IDCases. 2019;17:e00575. 10.1016/j.idcr.2019.e00575 31304090 PMC6599882

[3] van der Lelie H, Leverstein-Van Hall M, Mertens M, van Zaanen HC, van Oers RH, Thomas BL, et al. *Corynebacterium* CDC group JK (*Corynebacterium jeikeium*) sepsis in haematological patients: a report of three cases and a systematic literature review. Scand J Infect Dis. 1995;27:581–4. 10.3109/00365549509047071 8685637

[4] Lee PP, Ferguson DA Jr, Sarubbi FA. *Corynebacterium striatum*: an underappreciated community and nosocomial pathogen. J Infect. 2005;50:338–43. 10.1016/j.jinf.2004.05.005 15845432

[5] Bao R, Gao X, Hu B, Zhou Z. Matrix-assisted laser desorption ionization time-of-flight mass spectrometry: a powerful tool for identification of *Corynebacterium* species. J Thorac Dis. 2017;9:3239–45. 10.21037/jtd.2017.09.69 29221301 PMC5708486

[6] Rezaei Bookani K, Marcus R, Cheikh E, Parish M, Salahuddin U. *Corynebacterium jeikeium* endocarditis: A case report and comprehensive review of an underestimated infection. IDCases. 2017;11:26–30. 10.1016/j.idcr.2017.11.004 29619320 PMC5881414

[7] Ghide S, Jiang Y, Hachem R, Chaftari AM, Raad I. Catheter-related *Corynebacterium* bacteremia: should the catheter be removed and vancomycin administered? Eur J Clin Microbiol Infect Dis. 2010;29:153–6. 10.1007/s10096-009-0827-0 20016995

[8] Rasmussen M, Mohlin AW, Nilson B. From contamination to infective endocarditis-a population-based retrospective study of *Corynebacterium* isolated from blood cultures. Eur J Clin Microbiol Infect Dis. 2020;39:113–9. 10.1007/s10096-019-03698-6 31485919 PMC6962118

[9] Yanai M, Ogasawasa M, Hayashi Y, Suzuki K, Takahashi H, Satomura A. Retrospective evaluation of the clinical characteristics associated with *Corynebacterium* species bacteremia. Braz J Infect Dis. 2018;22:24–9. 10.1016/j.bjid.2017.12.002 29360429 PMC9425686

[10] Raad I, Hanna HA, Alakech B, Chatzinikolaou I, Johnson MM, Tarrand J. Differential time to positivity: a useful method for diagnosing catheter-related bloodstream infections. Ann Intern Med. 2004;140:18–25. 10.7326/0003-4819-140-1-200401060-00007 14706968

[11] Mermel LA, Allon M, Bouza E, Craven DE, Flynn P, O’Grady NP, et al. Clinical practice guidelines for the diagnosis and management of intravascular catheter-related infection: 2009 Update by the Infectious Diseases Society of America. Clin Infect Dis. 2009;49:1–45. 10.1086/599376 19489710 PMC4039170

[12] Horan TC, Andrus M, Dudeck MA. CDC/NHSN surveillance definition of health care-associated infection and criteria for specific types of infections in the acute care setting. Am J Infect Control. 2008;36:309–32. 10.1016/j.ajic.2008.03.002 18538699

[13] Hudspeth MK, Hunt Gerardo S, Citron DM, Goldstein EJ. Evaluation of the RapID CB Plus system for identification of *Corynebacterium* species and other gram-positive rods. J Clin Microbiol. 1998;36:543–7. 10.1128/JCM.36.2.543-547.1998 9466773 PMC104574

[14] Kanda Y. Investigation of the freely available easy-to-use software ‘EZR’ for medical statistics. Bone Marrow Transplant. 2013;48:452–8. 10.1038/bmt.2012.244 23208313 PMC3590441

[15] Ishiwada N, Watanabe M, Murata S, Takeuchi N, Taniguchi T, Igari H. Clinical and bacteriological analyses of bacteremia due to *Corynebacterium striatum.* J Infect Chemother. 2016;22:790–3. 10.1016/j.jiac.2016.08.009 27654073

[16] Kimura SI, Gomyo A, Hayakawa J, Akahoshi Y, Harada N, Ugai T, et al. Clinical characteristics and predictive factors for mortality in coryneform bacteria bloodstream infection in hematological patients. J Infect Chemother. 2017;23:148–53. 10.1016/j.jiac.2016.11.007 28011352

[17] Souza C, Faria YV, Sant’Anna LO, Viana VG, Seabra SH, Souza MC, et al. Biofilm production by multiresistant *Corynebacterium striatum* associated with nosocomial outbreak. Mem Inst Oswaldo Cruz. 2015;110:242–8. 10.1590/0074-02760140373 25946249 PMC4489456

[18] Kang SJ, Choi SM, Choi JA, Choi JU, Oh TH, Kim SE, et al. Factors affecting the clinical relevance of *Corynebacterium striatum* isolated from blood cultures. PLoS One. 2018;13:e0199454. 10.1371/journal.pone.0199454 29928059 PMC6013186

[19] Kwaszewska AK, Brewczyńska A, Szewczyk EM. Hydrophobicity and biofilm formation of lipophilic skin corynebacteria. Pol J Microbiol. 2006;55:189–93. 17338271

[20] Dragomirescu CC, Lixandru BE, Coldea IL, Corneli ON, Pana M, Palade AM, et al. Antimicrobial susceptibility testing for *Corynebacterium* species isolated from clinical samples in Romania. Antibiotics (Basel). 2020;9:31. 10.3390/antibiotics9010031 31963167 PMC7168242

[21] Soriano F, Zapardiel J, Nieto E. Antimicrobial susceptibilities of *Corynebacterium* species and other non-spore-forming gram-positive bacilli to 18 antimicrobial agents. Antimicrob Agents Chemother. 1995;39:208–14. 10.1128/AAC.39.1.208 7695308 PMC162510

[22] Kara Ö, Zarakolu P, Aşçioğlu S, Etgül S, Uz B, Büyükaşik Y, et al. Epidemiology and emerging resistance in bacterial bloodstream infections in patients with hematologic malignancies. Infect Dis (Lond). 2015;47:686–93. 10.3109/23744235.2015.1051105 26024284

[23] Rozdzinski E, Kern W, Schmeiser T, Kurrle E. *Corynebacterium jeikeium* bacteremia at a tertiary care center. Infection. 1991;19:201–4. 10.1007/BF01644945 1917029

[24] Stamm WE, Tompkins LS, Wagner KF, Counts GW, Thomas ED, Meyers JD. Infection due to *Corynebacterium* species in marrow transplant patients. Ann Intern Med. 1979;91:167–73. 10.7326/0003-4819-91-2-167 380432

[25] Shariff M, Aditi A, Beri K. *Corynebacterium striatum*: an emerging respiratory pathogen. J Infect Dev Ctries. 2018;12:581–6. 10.3855/jidc.10406 31954008

[26] Bechara C, Gousseff M, Passeron A, Podglajen I, Day N, Pouchot J, et al. *Corynebacterium jeikeium* pacemaker infection associated with antineutrophil cytoplasmic antibodies: a single positive blood culture could be sufficient for diagnosis. J Med Microbiol. 2011;60:249–51. 10.1099/jmm.0.023283-0 20965920

[27] Cazanave C, Greenwood-Quaintance KE, Hanssen AD, Patel R. *Corynebacterium* prosthetic joint infection. J Clin Microbiol. 2012;50:1518–23. 10.1128/JCM.06439-11 22337986 PMC3347109

